# The King-Devick test of rapid number naming for concussion detection: meta-analysis and systematic review of the literature

**DOI:** 10.2217/cnc.15.8

**Published:** 2015-09-10

**Authors:** Kristin M Galetta, Mengling Liu, Danielle F Leong, Rachel E Ventura, Steven L Galetta, Laura J Balcer

**Affiliations:** 1 Department of Neurology, University of Pennsylvania School of Medicine, 3400 Spruce Street, Philadelphia, PA 19104, USA; 2 Department of Population Health, NYU School of Medicine, 240 East 38th Street, 20th Floor, New York, NY 10016, USA; 3 Illinois College of Optometry, 3241 S Michigan Ave, Chicago, IL 60616, USA; 4 Department of Neurology, NYU School of Medicine, 240 East 38th Street, 20th Floor, New York, NY 10016, USA; 5 Department of Ophthalmology, NYU School of Medicine, 240 East 38th Street, 20th Floor, New York, NY 10016, USA; 6 Department of Biostatistics & Epidemiology, University of Pennsylvania School of Medicine, Philadelphia, PA 19104, USA

**Keywords:** concussion, King-Devick test, meta-analysis, rapid number naming, saccades, sports, vision

## Abstract

**Background:**

Vision encompasses a large component of the brain's pathways, yet is not represented in current sideline testing.

**Objectives:**

We performed a meta-analysis of published data for a vision-based test of rapid number naming (King-Devick [K-D] test).

**Studies & methods:**

Pooled and meta-analysis of 15 studies estimated preseason baseline K-D scores and sensitivity/specificity for identifying concussed versus nonconcussed control athletes.

**Result:**

Baseline K-D (n = 1419) showed a weighted estimate of 43.8 s (95% CI: 40.2, 47.5; *I*^2^ = 0.0%; p=0.85 – indicating very little heterogeneity). Sensitivity was 86% (96/112 concussed athletes had K-D worsening; 95% CI: 78%, 92%); specificity was 90% (181/202 controls had no worsening; 95% CI: 85%, 93%).

**Conclusion:**

Rapid number naming adds to sideline assessment and contributes a critical dimension of vision to sports-related concussion testing.

Concussion is defined as a complex pathophysiological process affecting the brain from an impulsive force transmitted to the head or from a direct blow to the head, face, neck or elsewhere on the body that results in a new neurological sign or symptom [1]. Increasing public awareness of the incidence of concussion, estimated at 4 million per year, and the possible long-term consequences on brain function are becoming a growing concern for participants in contact and collision sports [2]. Additionally, an increasing number of military personnel are returning from recent conflicts with blast-induced concussion and traumatic brain injury [3–5].

During concussion, linear and rotational accelerations of the brain occur relative to the skull producing pressure and shear forces throughout the brain tissue [6]. This may lead to tissue damage and diffuse axonal stretching, and in turn, lead to diffuse axonal injury, which can cause disruption of cortical and subcortical pathways producing neurobehavioral dysfunction [6,7]. Exposure to repetitive concussion or subconcussive impacts is now recognized as having possible long-term neurological consequences, including neurodegenerative disease [2,8–10]. The general lack of radiological signs after concussion on conventional MRI and computed tomography has often left medical professionals to rely primarily upon clinical signs and symptoms to diagnose concussion [11–16]. In the acute or sideline setting, there is a need for testing that can be quickly administered to help confirm the diagnosis of concussion – or more importantly, given the simple clinical definition, to force critical thinking regarding a potentially meaningful neurologic event.

The development of a range of sideline screening tests has occurred in response to the concussion epidemic. Sideline tools such as the Sports Concussion Assessment Tool, 3rd Edition (SCAT3) include a Symptom Checklist and the Standardized Assessment of Concussion (SAC). The SAC is a cognitive component of SCAT3 that has also been incorporated into the Military Acute Concussion Evaluation (MACE). To test balance, the SCAT3 includes a modified version of the Balance Error Scoring System (BESS) [1], while the Child-SCAT3 relies upon the more recently developed timed tandem gait test. While both balance and neuropsychological testings have been shown to be effective in the assessment of concussion, there remain opportunities for improving the ability to consistently capture all concussive events quickly [17–21]. Balance function can be affected by fatigue [21–23] and previous injury [18,24]. The SAC has largely been validated using earlier concussion definitions, which required alteration of mental status, loss of consciousness or post-traumatic amnesia [25,26]. As such, the reported sensitivities of these measures may have been greater than those that could result when applied using more broad definitions of concussion. Furthermore, since concussive symptoms often go unreported at the high school or collegiate levels, there is a need for sensitive and quantitative concussion tests that can be used to support clinical or lay-witness suspicion of an event [27,28]. Recent work has demonstrated that the addition of a rapid, simple vision-based performance test to cognitive and balance-based sideline tests enhances the ability to detect concussions at youth and collegiate levels of play [29,30].

The visual system is important in the diagnosis of concussion for a number of reasons (Figure 1) [31–33]. These pathways travel from the eyes to the visual cortex with extensive connections made with countless areas in the frontal, parietal and temporal lobes. Cortical areas engaged in saccadic function include the frontal eye fields, dorsolateral prefrontal cortex (DLPFC), supplementary motor area, posterior parietal cortex, middle temporal area and striate cortex [34–40]. These areas are responsible for the planning, initiation and execution of coordinated saccades such as those needed for reading and rapid number naming [36,41]. The DLPFC also plays a crucial role in the control of saccades through the suppression of unwanted eye movements (antisaccades) [37,40,42]. Other subcortical structures involved in eye movements include the thalamus, superior colliculus, cerebellum and other structures within the brainstem [41]. The interdependency of the neural activity between these areas illustrates the importance of their functional integrity for proper eye movement function. Additionally, these complex circuits involve cognitive processing such as memory, attention and language function [35]. Pathology at any of these various levels in a functional pathway will result in errors of performance [35]. Given that the network of visual and eye movement pathways is widely distributed throughout the brain, saccade testing is well suited to examine the neurophysiologic effects of concussion.

**Figure 1.**
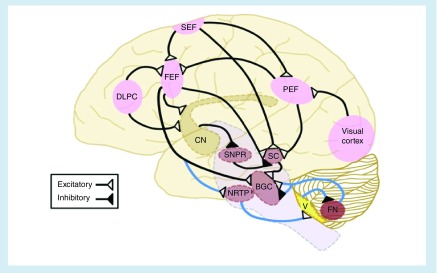
**Major cortical areas involved in control of eye movements and visual processing, with projections illustrating saccade generation in black.** Major cortical areas involved in control of eye movements and visual processing, with projections illustrating saccade generation in black. Saccades are initiated by signals sent from the frontal, parietal or supplementary eye fields to the superior colliculus, which then projects to the brainstem gaze centers. In parallel, the FEF also initiates saccades via direct connections to the BGC. In the indirect pathway, the substantia nigra pars reticulata inhibits the superior colliculus, preventing saccade generation. To turn off this inhibition, the FEFs are activated prior to a saccade, which then inhibits the substantia nigra pars reticulata via the caudate. The saccade pathways are a multidistributed network, but the FEF primarily generates voluntary- or memory-guided saccades, the parietal eye field – reflexive saccades, the SEF – saccades in coordination with body movements as well as successive saccades and the DLPC – antisaccades, the inhibition of reflexive saccades and the advanced planning of saccades. Cerebellar projections (shown in blue) fine-tune the saccades, given that cerebellar lesions can lead to saccadic dysmetria. The nucleus reticularis tegmenti pontis receives projections from the FEF and the superior colliculus (projection not shown) and in turn projects to the cerebellar ocular V. The Vinhibits the ipsilateral caudal fastigial nucleus, which then projects to the BGC to enhance saccades moving to the contralateral side and tamp down saccades moving to the ipsilateral side, likely via both inhibitory and excitatory connections [33,70,71]. BGC: Brainstem gaze centers; CN: Caudate; DLPC: Dorsolateral prefrontal cortex; FEF: Frontal eye field; FN: Fastigial nucleus; NRTP: Nucleus reticularis tegmenti pontis; PEF: Parietal eye fields; SC: Superior colliculus; SEF: Supplementary eye field; SNPR: Substantia nigra pars reticulata; V: Vermis.

The efferent visual pathways are particularly vulnerable to injury in the acute setting of concussion [34,43–46] and may be assessed through visual performance measures such as rapid number naming tasks. The K-D test is a two minute rapid number naming assessment in which an individual reads numbers aloud quickly from test cards or a computer-based application (Figure 2). The K-D test requires eye movements (saccades, convergence and accommodation), attention and language function. These tasks involve the integration of functions of the brainstem, cerebellum and cerebral cortex. Performance on the K-D test has been shown to correlate with suboptimal brain function in concussion [29,30,47–56], Parkinson's disease [57], multiple sclerosis (MS) [58], amyotrophic lateral sclerosis [59], sleep deprivation [60] and hypoxia [61,62]. Patients with Parkinson's disease and those with amyotrophic lateral sclerosis have been found to have slower (worse) K-D times than healthy controls [57,59]. In one study of astronaut trainees with experimentally induced hypoxia, mean K-D times were found to be 54.5 s compared with 46.3 s for controls (p = 0.02) [61]. Investigation of the K-D test in an MS cohort showed K-D scores that were significantly worse than disease-free controls, accounting for age (p < 0.001). Also in the MS study, higher (worse) K-D times were associated with worse vision-specific quality of life among MS patients as measured by the 25-Item National Eye Institute Visual Functioning Questionnaire (NEI-VFQ-25; p = 0.001) and correlated with measures of MS disability in MS including the timed 25-foot walk (p < 0.001) [58]. In a study of neurology residents, those taking call with an average of 2 h sleep had smaller magnitudes of learning effect from a precall baseline compared with participants who did not take call over a 24-h period (p < 0.0001, Wilcoxon rank sum test) [60].

**Figure 2.**
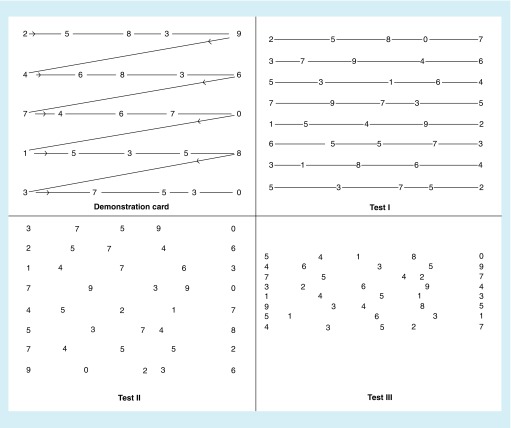
**Demonstration and test cards for the King-Devick test, a candidate rapid sideline screening for concussion based on speed of rapid number naming.** To perform the King-Devick test, participants are asked to read the numbers on each card from left to right as quickly as possible, but without making any errors. Following completion of the demonstration card (upper left), subjects are then asked to read each of the three test cards in the same manner. The times required to complete each card are recorded in seconds using a stopwatch. The sum of the three test card time scores constitutes the summary score for the entire test, the King-Devick time score. Numbers of errors made in reading the test cards are also recorded; misspeaks on numbers are recorded as errors only if the subject does not immediately correct the mistake before going on to the next number.

In the setting of concussion, the K-D test has been studied across several cohorts and throughout a variety of contact sports, including boxers and mixed martial arts (MMA) fighters, collegiate athletes in contact sports, amateur rugby players, elite professional hockey players, high school level football and youth contact sport athletes [29,30,47–56]. The purpose of this investigation was to perform a meta-analysis and systematic review of all studies of the K-D test across athletic event types, and to summarize the literature examining the K-D test as a rapid sideline tool to aid in the detection of concussion.

## Studies & methods

Following generally accepted methodologic recommendations, these pooled and meta-analyses were performed according to the PRISMA (Preferred Reporting Items for Systematic Review and Meta-Analyses) statement [63]. This checklist contains specifications for the selection and review of studies for inclusion in the meta-analysis.

### Search strategy

**Figure 3.**
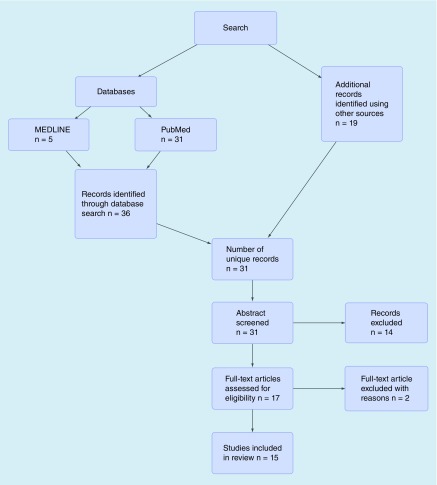
**Study selection process for pooled and meta-analyses.**

The systematic literature search was begun in January 2015 and concluded in April 2015. Two independent investigators searched for relevant articles. Each investigator searched PubMed and MEDLINE using specific key search terms including: ‘King Devick’, ‘concussion’, ‘sideline concussion test’ and ‘concussion assessment’. Additional studies were identified by checking reference lists of the retrieved studies, searching clinical trial registries and contacting clinical experts. PubMed searches yielded 31 results and MEDLINE revealed five articles (Figure 3). After screening for duplicates, there were 31 manuscripts. Articles must have included the K-D test as a baseline or postinjury measure for detecting concussion, and have been accepted for publication in a peer-reviewed journal. Abstracts were screened for those studies that met criteria, and this left 17 articles eligible for consideration of inclusion in the meta-analysis. Two published studies were excluded because investigations were ongoing, prohibiting analyses as part of the present study. Thus, there were 15 studies (14 published texts and an article accepted for publication) included in these pooled and meta-analyses.

### Study selection

The algorithm by which studies were identified for inclusion is shown in Figure 3. All English language published studies including data and examining the use of the K-D test for concussion from 2010 to present were included in these analyses. Publications were included if they met the following criteria:

- The study participants were athletes;
- Deidentified, participant-specific data were available for pooled analyses;
- The study examined the K-D test in the setting of sports-related concussion with baseline measurements (testing time from injury was available);
- The study defined concussion by the standard definition of a witnessed or reported blow to the head or body followed by new neurological signs or symptoms;
- Concussions, when captured by the study, occurred within the context of a sporting event.

Eligibility assessments were performed independently by two reviewers. Disagreements regarding study inclusion were discussed between the reviewers; it was planned that any further disagreements would be mediated by a third investigator. Participant-specific data, with all potential identifiers removed, were obtained for analyses that were performed at the NYU School of Medicine. Questions regarding potential for duplicate publication of specific data were clarified with the authors of each study.

### Data items & collection

Data were pooled into a single dataset with a variable representing an identification number for each study. Variables retained in the pooled dataset included: nonidentifying characteristics of participants; preseason or prematch baseline K-D test scores, in seconds; other baseline concussion test scores (not available for all participants), including components of the Sport Concussion Assessment Tool (SCAT; 2nd and 3rd editions); postinjury K-D test scores following concussion (not available for all participants) and for nonconcussed control athletes of similar age, gender and sport; K-D test scores following vigorous workout or match play in the absence of concussion (fatigue trial testing, not available for all participants); and postseason K-D test scores (not available for all participants).

### Assessment of bias

Two reviewers worked independently to determine the validity of studies included in these analyses with regard to data collectors and outcome assessors. Some studies whose data were included were designed and executed by authors of the present study. Different study designs were represented, for example, some studies included nonconcussed control athletes who underwent K-D testing in parallel with concussed athletes for comparison of performance. The definitions of concussion and control athlete were consistent throughout the studies represented in the pooled dataset. Assessments and K-D testing were performed in all studies at the time of first recognition of a concussive event or symptoms.

There are other potential risks of bias inherent to pooled and meta-analyses in general. Language bias was not a particular risk for our study since all published data and manuscripts were in English. Availability bias was avoided through the use of a variety of techniques to obtain articles (see Search strategy). All studies were included regardless of the direction and statistical significance of results comparing concussed versus nonconcussed control athletes.

### Data analyses

The primary objectives of these pooled and meta-analyses included the following:

- To calculate pooled and weighted estimates and measures of precision (means and 95% CIs) for preseason/prematch baseline K-D test scores across the 15 studies;
- To calculate pooled and weighted estimates and measures of precision (mean and 95% CIs) for changes in K-D score from baseline at the time of postinjury recognition of concussion among those studies with postconcussion data included in the pooled dataset;
- To perform similar analyses to #2 above for nonconcussed control athletes among studies with control data included in the pooled dataset;
- To estimate sensitivity and specificity of K-D test score worsening (defined as any increase in time score from baseline) for identifying concussed versus nonconcussed control athletes using pooled analysis techniques;
- To calculate the weighted relative risk of an athlete being concussed versus a nonconcussed control in the setting of K-D score worsening from preseason/prematch baseline using meta-analysis with fixed effects models.

Stata 13.1 (StataCorp, TX, USA) software was used to perform all statistical analyses and calculations. Data for 15 studies with deidentified participant-specific values were analyzed. Pooled analyses estimated preseason baseline K-D scores, participants age and sensitivity/specificity for K-D to identify concussed versus control athletes on the sidelines (athletes who were playing the game but did not have concussion). Fixed-effects models were used for meta-analysis to calculate weighted estimates in objectives 1–3 and 5. Heterogeneity across studies in the meta-analysis was evaluated using the *I*^2^ (I-squared) statistic. *I*^2^ is the percentage of variation in a pooled dataset that is attributable to heterogeneity between studies (systematic differences). A value of 0% indicates no observed heterogeneity, and larger values show increasing heterogeneity. While there is no absolute rule for when heterogeneity becomes important, authorities in the field [64] have suggested categories of low heterogeneity for *I*^2^ values between 25 and 50%, moderate for 50–75%, and high for 75% or greater. Thus, in the case of the *I*^2^ statistic, low percentages and high p-values are the desired result.

Areas under the receiver operating characteristic (ROC) curves generated from logistic regression models were estimated from the pooled dataset to examine the capacity for K-D scores to distinguish concussed versus nonconcussed control athletes based on changes from preseason or prematch baseline. The area under the ROC represents the discriminatory ability of a continuous test score to correctly classify any two individuals in a study with and without the disease (in this case, concussed vs nonconcussed). These areas range from 0.50 (indicating no better ability to distinguish than the flip of a fair coin) to 1.0 (perfect ability to distinguish).

## Results

**Figure 4.**
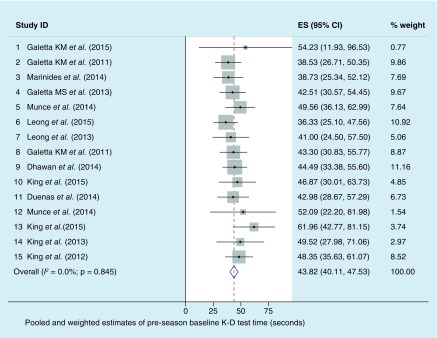
**Distribution of preseason baseline time scores for the King-Devick test.** Dots represent point estimates of each study mean (or ES); sizes of the gray boxes reflect the weights of the studies in the meta-analysis. Bars are 95% CI. The diamond shows the weighted estimate for the mean preseason K-D baseline score; this is determined from fixed-effects models account for study N and precision (narrowness of 95% CI). *I*^2^ statistic values were 0.0%; p = 0.85; indicating very little heterogeneity between studies in calculation of the weighted estimate. Stated differently, the nonsignificance of the *I*^2^ test for heterogeneity suggests that the differences between the studies are explicable by random variation rather than systematic factors. ES: Effect size; K-D: King-Devick. Data taken from [29,30,47,48,49,50,51,52,53,54,55,56,72,73,74].

Preseason baseline K-D scores from 1419 youth, collegiate, amateur and professional athletes were analyzed from among the 15 studies included in the pooled dataset. Study-specific characteristics of the athletes are presented in Table 1. Mean age from pooled analyses of the overall athlete cohort (all studies combined without weighting) was 18.3 years (95% CI: 18.0, 18.7), range 5–63. The weighted estimate of age from the meta-analysis was 14.8 years (95% CI: 14.1, 15.4). Pooled analysis means preseason baseline K-D scores were 44.5 s (95% CI: 43.8, 45.2), with a weighted estimate of 43.8 s (95% CI: 40.2, 47.5). *I*^2^ (I-squared) values were 0.0%, p = 0.85, indicating very little heterogeneity between studies in calculation of the weighted estimate (Figure 4). Stated differently, the nonsignificance of the *I*^2^ test for heterogeneity suggests that the differences between the studies are explicable by random variation rather than systematic factors.

**Table 1.** **Study characteristics and baseline rapid number naming (King-Devick) scores.**

**Study ID and reference**	**n**	**Age at Preseason baseline, years (95% CI)**	**K-D test preseason baseline score, seconds (95% CI)**	**Gender**	**Level of play**	**Sport**	**Ref.**
1. Galetta KM *et al*. (2015)	312	13.3 (12.8, 13.8)	54.2 (51.8, 56.6)	M/F	Youth/college	Hockey, lacrosse	[29]

2. Galetta KM *et al*. (2011)	217	20.3 (20.1, 20.5)	38.5 (37.7, 39.3)	M/F	College	Football, basketball	[48]

3. Marinides *et al*.(2014)	220	–	38.7 (37.8, 39.6)	M/F	College	Football, lacrosse, soccer	[30]

4. Galetta MS *et al*. (2013)	69	24.5 (23.2, 25.8)	42.5 (41.0, 43.9)	M	Professional	Hockey	[50]

5. Munce *et al*. (2014)	15	13.3 (12.9, 13.6)	49.6 (45.7–53.4)	M	Youth	Football	[72]

6. Leong *et al*. (2015)	152	19.6 (19.4, 19.8)	36.3 (35.4, 37.3)	M/F	College	Football, basketball	[53]

7. Leong *et al*. (2014)	34	25.8 (22.9, 28.6)	41.0 (38.1, 43.9)	M/F	Amateur	Boxing	[52]

8. Galetta KM *et al*. (2011)	42	27.2 (24.2, 30.1)	43.3 (41.3, 45.3)	M	Amateur	Boxing	[47]

9. Dhawan *et al*. (2014)	140	15.5 (15.3, 15.7)	44.5 (43.5, 45.4)	M	Youth	Hockey	[54]

10. King *et al*. (2015)	68	24.5 (23.4 25.7)	46.9 (44.8, 48.9)	M	Amateur	Rugby	[56]

11. Duenas *et al*. (2014)	12	16.5 (15.8, 17.2)	43.0 (38.3, 47.6)	M	Youth	Football	[55]

12. Munce *et al*. (2014)	22	12.8 (12.5, 13.0)	52.1 (45.3, 58.9)	M	Youth	Football	[73]

13. King *et al*. (2015)	19	–	62.0 (57.2, 66.7)	M/F	Youth	Rugby	[74]

14. King *et al*. (2013)	36	23.5 (22.1, 24.8)	49.5 (45.8, 53.2)	M	Amateur	Rugby	[49]

15. King *et al*. (2012)	61	19.3 (18.3, 20.3)	48.4 (46.7, 50.0)	M	Amateur	Rugby	[51]

K-D: King-Devick; M: Male; F: Female; n: Number of athletes

**Figure 5.**
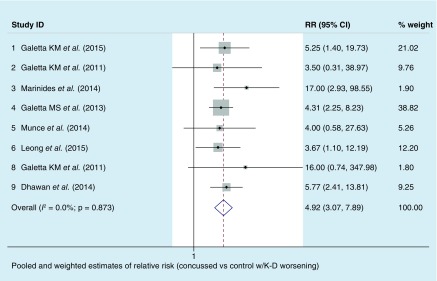
**Distribution of relative risk of concussed versus control athlete status in the setting of any worsening of time score from preseason baseline for the King-Devick test.** Dots represent point estimates of each study's relative risk; size of the gray box reflects the weight of the study in meta-analysis. Bars are 95% CI. The diamond shows the weighted estimate for the relative risk; this is determined from fixed-effects models account for study N and precision (narrowness of 95% CI). *I*^2^ statistic values were 0.0%; p = 0.87, indicating very little heterogeneity between studies in calculation of the weighted estimate. Stated differently, the nonsignificance of the *I*^2^ test for heterogeneity suggests that the differences between the studies are explicable by random variation. Study ID numbers are not consecutive since some studies did not have postinjury data. RR: Relative risk; K-D: King-Devick. Data taken from [29,30,47,48,50,53,54,72].

Among 112 athletes with concussion in the dataset, weighted estimates for postinjury changes in K-D score from preseason or prematch baseline showed a worsening (increased time) of 4.8 s (95% CI: 3.7, 5.8; *I*^2^ = 0.0%; p = 0.58 – large p-value indicating very little heterogeneity and good consistency between studies); nonconcussed control athletes demonstrated an improvement of 1.9 s (95% CI: -3.6 to -0.02; *I*^2^ = 0.0%; p = 0.99). Pooled analysis values for sensitivity of the K-D test for detecting concussion on the sidelines were 86% (96/112 concussed athletes had any worsening of K-D time score from baseline; 95% CI: 78%, 92%); specificity was 90% (18 1/2 02 nonconcussed control athletes had no worsening of K-D from baseline, 95% CI: 85%, 93%). Relative risk from meta-analysis of these proportions across studies was 4.92 (95% CI: 3.07, 7.89; *I*^2^ = 0.0%; p = 0.87), indicating an approximately five-times greater likelihood of a participant being a concussed versus a control athlete, if their K-D score worsened from preseason or prematch baseline (Figure 5). Increasing age was significantly associated with decreasing K-D time scores in linear regression models for the pooled dataset.

**Figure 6.**
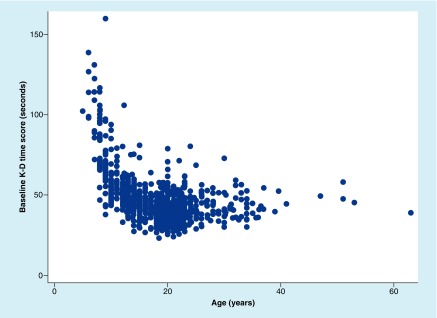
**Relation of rapid number naming (King-Devick) scores to athlete age.** The scores improve (are faster) with age among youth (age 18 years and younger), then appear stable with perhaps some increase by the end of the fourth decade. K-D: King-Devick.

Similar to recently published studies reporting times for the three K-D test cards, baseline scores for each card were lowering (faster) with increasing age in this cohort of predominantly young athletes (p < 0.001, linear regression models). This age effect was particularly evident for K-D test card 3, which has the greatest degree of vertical crowding of the numbers (Figure 2; p < 0.001 for magnitude of correlation of score with age for test card 3 vs 1). Furthermore, total time scores improved (are faster) overall with age among youth (age 18 and younger), then appeared stable with perhaps some increase by the end of the fourth decade (Figure 6).

**Figure 7.**
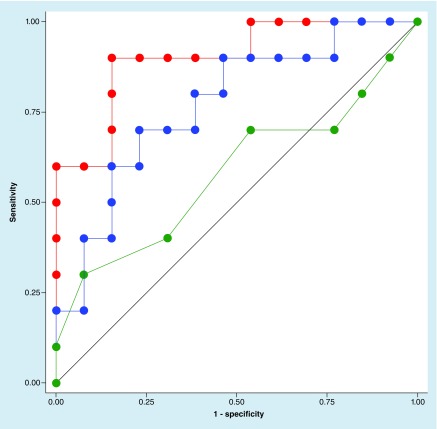
**Comparisons of receiver operating characteristic curve areas for the three sideline tests for distinguishing concussed versus nonconcussed control athletes on the sideline among athletes who underwent all measures (n = 23).** King-Devick = red line versus timed tandem gait = blue line versus Standardized Assessment of Concussion = green line. Receiver operating characteristic curve areas represent the probability that a test or combination can correctly distinguish between two categories (concussed vs nonconcussed control).

Using the pooled dataset, the capacity for K-D scores to distinguish the concussed (n = 112) versus nonconcussed control athletes (n = 202) based on changes from preseason or prematch baseline was examined using areas under ROC curves generated from logistic regression models. The ROC area was 0.90 (95% CI: 0.85, 0.96), indicating an approximately 90% chance of correctly distinguishing concussed versus control athlete status on the basis of change in K-D score from baseline alone. Accounting simultaneously for age, these same models yielded an ROC curve area of 0.89 (95% CI: 0.82, 0.96; n = 239 total participants with age recorded). Among participants who underwent testing with the SAC and timed tandem gait components of SCAT in addition to K-D (n = 69), ROC areas, from models accounting for age, were 0.89 for K-D (95% CI: 0.82, 0.96), 0.81 for timed tandem gait (95% CI: 0.69, 0.92) and 0.66 for SAC (95% CI: 0.53, 0.79; Figure 7). These differences in ROC curves were significant (p = 0.002, by linear combination methods). Using the criteria of any worsening of score from baseline for K-D, and published criteria of two-point worsening for SAC and any worsening for timed tandem gait score, the K-D worsened from baseline in 85% of concussed athletes in the pooled dataset. SAC worsened in 48% and tandem worsened in 75%; worsening of either SAC or timed tandem was observed in 89%. Importantly, however, worsening of at least one of the three tests was observed in 100% of concussed athletes, supporting the capacity for a composite of tests to capture concussions to a greater degree than tests of a single neurologic dimension.

Similar to many performance measures, the K-D test demonstrated a mild learning effect when administered over two trials at the preseason baseline. For participants with two K-D baseline trials (n = 1048), the pooled analysis mean for the first trial was 49.5 s (95% CI: 48.6, 50.4); the second trial had a slightly faster time score of 46.7 s (95% CI: 45.8, 47.6; p < 0.0001, paired t-test). This translates into a learning effect of 2.8 s improvement on average between the two baseline trials (95% CI: -3.1, -2.1) in analyses of the pooled dataset. In the meta-analysis, the weighted mean improvement was 1.8 s (95% CI: -3.4; -0.1, *I*^2^ = 0.0%, p = 0.98). Despite the inherent and expected learning effects for the K-D in the absence of concussion, test-retest reliability was very high between the two baseline trials, with intraclass correlation coefficient values (ICC) = 0.92 (95% CI: 0.91, 0.94). This indicates that 92% of the variability in the dataset is between participants, rather than between the two K-D baseline trials from the same participants.

K-D scores also improved following vigorous exercise in the absence of concussion. On average in the pooled dataset, K-D scores were 1.4 s better (95% CI: -2.1, -0.8) than the preseason baseline among 92 participants who underwent testing immediately following active competition or vigorous exercise. The weighted estimate from the three studies with postexercise data was similar, showing an improvement of 1.2 s (95% CI: -4.3, 1.8; *I*^2^ = 0.0%; p = 0.52). Test-retest reliability in this setting was likewise high, with ICC = 0.91 (95% CI: 0.85, 0.97).

## Discussion

Rapid number naming (K-D test), a timed vision-based measure that requires the integration of eye movements, number identification, demonstrates high sensitivity and specificity in distinguishing concussed athletes from controls. The test has been investigated across a variety of contact sports, including boxing and MMA, collegiate athletics, amateur rugby, professional hockey, high school level football and youth sports [29,30,47–56]. Collectively, these studies and the meta-analyses reported herein demonstrate that worsening of K-D scores from a preseason or prematch baseline is an accurate and sensitive indicator of concussion and emphasizes the importance of comparison with an individualized baseline, not normative data. In these meta-analyses, concussed athletes showed a mean worsening from baseline of 4.8 s (95% CI: 3.7, 5.8; *I*^2^ = 0.0%; p = 0.58 – large p-value indicating very little heterogeneity and good consistency between studies) while nonconcussed control athletes demonstrated an improvement of 1.9 s (95% CI: -3.6, -0.02; *I*^2^ = 0.0%; p = 0.99). These weighted means, derived from multiple independent investigations, are perhaps most illustrative of the concept that any worsening of K-D score from baseline is suggestive that a meaningful neurological event has occurred.

Test-retest reliability of K-D has been investigated in multiple studies. High levels of reliability have been reported in the absence of concussion with ICC's ranging from 0.95 (95% CI: 0.87, 1.0) to 0.97 (95% CI: 0.90, 1.0) in studies of boxers and MMA fighters [47]. ICC values of 0.95 (95% CI: 0.85, 1.1) were noted among collegiate athletes [52]. In a study examining how well sports parents could reliably administer the K-D test in boxers, equally high levels of test-retest metrics were observed (ICC = 0.90; 95% CI: 0.84, 0.97) [52]. Collectively, these ICC values demonstrate that both medical personnel and laypersons may administer the K-D test with high degrees of reliability. Our current meta-analysis similarly demonstrated high test-retest reliability in the absence of head trauma (ICC = 0.92; 95% CI: 0.91, 0.94). The use of the K-D test in combination with other sideline tests has been investigated. Marinides *et al*. [30] showed that among 30 athletes with concussion, worse scores on the K-D occurred in 79%, while worsening of the SAC by two points was noted in 52% of concussed athletes. When the K-D and SAC were combined, their ability to detect concussion was increased to 89%, and increased to 100% when the BESS was added. In 2015, a study of the K-D test in ice hockey and lacrosse athletes at the youth and collegiate levels examined the SAC, timed tandem gait and K-D test on the sidelines and rinkside for concussed athletes as well as nonconcussed controls under the same testing conditions. Among athletes who sustained concussion (n = 12), K-D scores worsened from baseline by an average of 5.2 s. In contrast, nonconcussed control athletes improved on average by 6.4 s. In comparing the SAC, timed tandem gait and K-D test with regard to capacity to distinguish concussed athletes versus nonconcussed controls (based on changes from preseason baseline to postinjury), ROC curve areas were 0.92 for K-D, 0.87 for timed tandem gait and 0.68 for SAC (p = 0.0004 for comparison of ROC curve areas from logistic regression models, accounting for athlete age) [29].

The association of age on K-D time score was also explored in a recent study [29] in which scores decreased (improved) with advancing age among youth athletes. In particular, the youngest athletes within the 5–18-year old range had significantly slower K-D times (p < 0.001) and demonstrated, as in our cohort, the greatest improvements in score occur from ages 5 to approximately 18 years (Figure 6). These differences in time could potentially be explained by the brain development of youth athletes; diffusion tensor imaging MRI studies show that both white matter and grey matter changes continue in the frontal lobes throughout childhood and eye movement tasks required to perform the K-D test involve frontal eye field circuits [65]. Published literature demonstrates that saccade performance improves with development during childhood and that this is largely due to shortening of saccade reaction times, or latency. Not simply brainstem mediated (brainstem execution of saccades is stable throughout childhood), this phenomenon has been shown by functional MRI to be directly related to stronger activation in cortical eye fields, which enhances saccade preparation [66,67]. The K-D may well be capturing these developmental changes. The increased vertical crowding that characterizes the number stimuli of K-D test card 3 may also contribute to the slower time scores among younger children. In this meta-analysis, age was also significantly associated with K-D times. Taken together, these findings support the need to perform K-D preseason baselines atleast annually, if not every playing season, among young athletes.

The Developmental Eye Movements test, which involves a very similar paradigm to the K-D test and involves vertical saccades, has also been studied as a diagnostic tool [68], but its use in concussion assessment has been limited. A number of new metrics and automated portable devices that can detect eye movements and other visual abnormalities have also been developed [69]. These tools can be used in conjunction with helmet mounting or with telemedicine to enable sideline detection of signs of concussion.

Effects of the testing environment on K-D test scores have been investigated in different studies. One important question is the potential for vigorous exercise to affect performance on the K-D test. As captured in these pooled and meta-analyses, data have demonstrated that competition alone does not worsen K-D scores from baseline, but is rather associated with the same mild learning effects that are observed in studies of test-retest reliability. In the absence of concussion, the present study as well as published investigations showed an average improvement of 1.4 s in the setting of vigorous exercise [48,49,53]. Other environmental factors, such as noise in the testing area, have also been examined. In a small pilot study in which participants (n = 9) completed the K-D test in a quiet environment and within two loud environments (with speakers and headphones), no significant difference in K-D scores was found between the testing conditions. Further, baseline testing of athletes in some of the studies [48] occurred in a busy training room environment. Improvement in K-D times among nonconcussed control athletes within the noisy game setting also suggests that K-D performance is relatively resistant to test conditions.

Construct validity of the K-D test as an instrument to capture meaningful neurologic events, such as concussion, has been explored with formal computerized eye movement recordings of individuals with hypoxia-induced impairment. In these studies, worsening of K-D performance was associated with changes in quantitative eye movement metrics [61,62]. Furthermore, performance on other cognitive measures such as the SAC and a similar tool called the MACE has correlated with K-D test outcomes both at baseline and postinjury [29,30,47,48,50]. These associations are likely due to shared anatomical aspects engaged in the execution of eye movements required for both the K-D and the SAC/MACE, such as predictive saccades and immediate memory. The DLPFC, for example, is one such structure that is also vulnerable to injury in concussion (Figure 1). To the extent that the SCAT3 does not assess vision and eye movements, and that K-D test scores are better among those with higher scores on the SAC and MACE, these findings likely explain why the K-D complements cognitive sideline testing [30]. In these studies of collegiate level athletes, 10% of concussed athletes failed to be shown as significant on SAC and BESS. Addition of the K-D test, however, captured all of the concussions. Similar findings were noted in the pooled dataset of the present study.

Future studies of the K-D test will include examining the underlying mechanisms and nature of eye movements during the rapid number naming task through quantitative electronic eye movement recordings. Evaluation of the capacity of K-D to capture functional improvement during longitudinal recovery following concussion will be important, and will increase our understanding of the timing of visual pathway recovery over time.

## Conclusion & future perspective

This meta-analysis demonstrates that preseason baseline scores are consistent across published studies, with high degrees of precision and little heterogeneity by meta-analytic techniques. The K-D test detects concussion with high degrees of sensitivity and specificity, with any worsening of time score from baseline, indicating a five-times greater likelihood of concussion. Test-retest reliability is high, and vigorous exercise alone is associated with mild learning effects rather than worsening of scores from preseason baseline. Among youth, collegiate and adult amateur and professional athletes, rapid number naming using the K-D test adds significantly to sideline assessment and contributes a critical dimension of vision to sports-related concussion testing.

**Executive summary**

- There is a concussion epidemic among athletes, and this extends to military personnel and other population groups.
- Given the widespread distribution of the visual pathways throughout the brain, a sideline test incorporating vision may aid in the diagnosis of concussion.
- The King-Devick (K-D) test, a rapid vision-based performance measure of rapid number naming, has been examined in a range of athletes at different ages and may be useful in the identification of concussion.
- Any worsening of baseline K-D test time at the time of an injury indicates a 5× greater risk of concussion.

**Reliability & validity of the K-D test**

- The K-D test was found to be reliable when administered by both trained personnel and laypersons.
- The K-D test was found to be both sensitive (86%) and specific (90%) for the detection of concussion.

**A sideline testing composite**

- The use of K-D along with the Standardized Assessment of Concussion and Balance Error Scoring System has been shown to detect 100% of clinically diagnosed concussions in this meta-analysis.
- The addition of a vision-specific concussion test to currently recommended concussion screening tools may expand the ability to detect concussion.

**Age & the K-D test**

- Baseline K-D times have been shown to be improved (decrease) with increasing age among youth athletes.
- Baseline K-D tests should be performed at least seasonally.

**Environment & K-D test**

- Noise has not been shown to significantly impact K-D times.
- Fatigue or competition alone does not to impact K-D times; in fact, time scores often improve after vigorous exercise.

**Conclusion & future perspective**

- The K-D test is a rapid, reliable, sensitive and specific test for concussion. Any worsening in time from a baseline K-D score is indicative of a concussion. The K-D test has the potential to screen for unwitnessed, or sub-concussive neurologic impairment as the result of injury from impulsive forces.

## References

[1] McCrory P, Meeuwisse WH, Aubry M (2013). Consensus statement on concussion in sport: the 4th International Conference on Concussion in Sport held in Zurich, November 2012. *Br. J. Sports Med.*.

[2] Plassman BL, Havlik RJ, Steffens DC (2000). Documented head injury in early adulthood and risk of Alzheimer's disease and other dementias. *Neurology*.

[3] Eibner C, Schell TL, Jaycox LH (2009). Care of war veterans with mild traumatic brain injury. *N. Engl. J. Med.*.

[4] Xydakis MS, Robbins AS, Grant GA (2008). Mild traumatic brain injury in U.S. soldiers returning from Iraq. *N. Engl. J. Med.*.

[5] Walsh DV, Capó-Aponte JE, Jorgensen-Wagers K (2015). Visual field dysfunctions in warfighters during different stages following blast and nonblast mTBI. *Mil. Med.*.

[6] Meaney DF, Smith DH (2011). Biomechanics of concussion. *Clin. Sports Med.*.

[7] Kraus MF, Susmaras T, Caughlin BP, Walker CJ, Sweeney JA, Little DM (2007). White matter integrity and cognition in chronic traumatic brain injury: a diffusion tensor imaging study. *Brain*.

[8] Gavett BE, Stern RA, McKee AC (2011). Chronic traumatic encephalopathy: a potential late effect of sport-related concussive and subconcussive head trauma. *Clin. Sports Med.*.

[9] Gavett BE, Cantu RC, Shenton M (2011). Clinical appraisal of chronic traumatic encephalopathy: current perspectives and future directions. *Curr. Opin. Neurol.*.

[10] Baugh CM, Stamm JM, Riley DO (2012). Chronic traumatic encephalopathy: neurodegeneration following repetitive concussive and subconcussive brain trauma. *Brain Imaging Behav.*.

[11] Bazarian JJ, Zhong J, Blyth B, Zhu T, Kavcic V, Peterson D (2007). Diffusion tensor imaging detects clinically important axonal damage after mild traumatic brain injury: a pilot study. *J. Neurotrauma*.

[12] Inglese M, Makani S, Johnson G (2005). Diffuse axonal injury in mild traumatic brain injury: a diffusion tensor imaging study. *J. Neurosurg.*.

[13] Hughes DG, Jackson A, Mason DL, Berry E, Hollis S, Yates DW (2004). Abnormalities on magnetic resonance imaging seen acutely following mild traumatic brain injury: correlation with neuropsychological tests and delayed recovery. *Neuroradiology*.

[14] Iverson GL, Lovell MR, Smith S, Franzen MD (2000). Prevalence of abnormal CT-scans following mild head injury. *Brain Inj.*.

[15] Shenton ME, Hamoda HM, Schneiderman JS (2012). A review of magnetic resonance imaging and diffusion tensor imaging findings in mild traumatic brain injury. *Brain Imaging Behav.*.

[16] Scheid R, Preul C, Gruber O, Wiggins C, Cramon von DY (2003). Diffuse axonal injury associated with chronic traumatic brain injury: evidence from T2*-weighted gradient-echo imaging at 3 T. *Am. J. Neuroradiol.*.

[17] Guskiewicz KM, McCrea M, Marshall SW (2003). Cumulative effects associated with recurrent concussion in collegiate football players: the NCAA Concussion Study. *JAMA*.

[18] Guskiewicz KM, Ross SE, Marshall SW (2001). Postural stability and neuropsychological deficits after concussion in collegiate athletes. *J. Athl. Train.*.

[19] Cavanaugh JT, Guskiewicz KM, Giuliani C, Marshall S, Mercer V, Stergiou N (2005). Detecting altered postural control after cerebral concussion in athletes with normal postural stability. *Br. J. Sports Med.*.

[20] Cavanaugh JT, Guskiewicz KM, Giuliani C, Marshall S, Mercer VS, Stergiou N (2006). Recovery of postural control after cerebral concussion: new insights using approximate entropy. *J. Athl. Train.*.

[21] Fox ZG, Mihalik JP, Blackburn JT, Battaglini CL, Guskiewicz KM (2008). Return of postural control to baseline after anaerobic and aerobic exercise protocols. *J. Athl. Train.*.

[22] Lepers R, Bigard AX, Diard JP, Gouteyron JF, Guezennec CY (1997). Posture control after prolonged exercise. *Eur. J. Appl. Physiol. Occup. Physiol.*.

[23] Thomas JR, Cotten DJ, Spieth WR, Abraham NL (1975). Effects of fatigue on stabilometer performance and learning of males and females. *Med. Sci. Sports*.

[24] Nardone A, Tarantola J, Giordano A, Schieppati M (1997). Fatigue effects on body balance. *Electroencephalogr. Clin. Neurophysiol.*.

[25] Barr WB, McCrea M (2001). Sensitivity and specificity of standardized neurocognitive testing immediately following sports concussion. *J. Int. Neuropsychol. Soc.*.

[26] McCrea M, Barr WB, Guskiewicz K (2005). Standard regression-based methods for measuring recovery after sport-related concussion. *J. Int. Neuropsychol. Soc.*.

[27] Register-Mihalik JK, Guskiewicz KM, McLeod TCV, Linnan LA, Mueller FO, Marshall SW (2013). Knowledge, attitude, and concussion-reporting behaviors among high school athletes: a preliminary study. *J. Athl. Train.*.

[28] Torres DM, Galetta KM, Phillips HW (2013). Sports-related concussion: anonymous survey of a collegiate cohort. *Neurol. Clin. Pract.*.

[29] Galetta KM, Morganroth J, Moehringer N (2015). Adding vision to concussion testing: a prospective study of sideline testing in youth and collegiate athletes. *J. Neuroophthalmol.*.

[30] Marinides Z, Galetta KM, Andrews CN (2015). Vision testing is additive to the sideline assessment of sports-related concussion. *Neurol. Clin. Pract.*.

[31] Felleman DJ, Van Essen DC (1991). Distributed hierarchical processing in the primate cerebral cortex. *Cereb. Cortex.*.

[32] Ventura RE, Jancuska JM, Balcer LJ, Galetta SL (2015). Diagnostic tests for concussion: is vision part of the puzzle?. *J. Neuroophthalmol.*.

[33] Ventura RE, Balcer LJ, Galetta SL (2014). The neuro-ophthalmology of head trauma. *Lancet Neurol.*.

[34] Heitger MH, Jones RD, Macleod AD, Snell DL, Frampton CM, Anderson TJ (2009). Impaired eye movements in post-concussion syndrome indicate suboptimal brain function beyond the influence of depression, malingering or intellectual ability. *Brain.*.

[35] White OB, Fielding J (2012). Cognition and eye movements: assessment of cerebral dysfunction. *J. Neuroophthalmol.*.

[36] Sparks DL, Mays LE (1990). Signal transformations required for the generation of saccadic eye movements. *Annu. Rev. Neurosci.*.

[37] Pierrot-Deseilligny C, Rivaud S, Gaymard B, Agid Y (1991). Cortical control of reflexive visually-guided saccades. *Brain*.

[38] Pierrot-Deseilligny C, Rivaud S, Gaymard B, Müri R, Vermersch AI (1995). Cortical control of saccades. *Ann. Neurol.*.

[39] Rivaud S, Müri RM, Gaymard B, Vermersch AI, Pierrot-Deseilligny C (1994). Eye movement disorders after frontal eye field lesions in humans. *Exp. Brain Res.*.

[40] Ploner CJ, Rivaud-Péchoux S, Gaymard BM, Agid Y, Pierrot-Deseilligny C (1999). Errors of memory-guided saccades in humans with lesions of the frontal eye field and the dorsolateral prefrontal cortex. *J. Neurophysiol.*.

[41] Heitger MH, Anderson TJ, Jones RD (2002). Saccade sequences as markers for cerebral dysfunction following mild closed head injury. *Prog. Brain Res.*.

[42] Gaymard B, Ploner CJ, Rivaud-Péchoux S, Pierrot-Deseilligny C (1999). The frontal eye field is involved in spatial short-term memory but not in reflexive saccade inhibition. *Exp. Brain Res.*.

[43] Goodrich GL, Flyg HM, Kirby JE, Chang C-Y, Martinsen GL (2013). Mechanisms of TBI and visual consequences in military and veteran populations. *Optom. Vis. Sci.*.

[44] Ciuffreda KJ, Kapoor N, Rutner D, Suchoff IB, Han ME, Craig S (2007). Occurrence of oculomotor dysfunctions in acquired brain injury: a retrospective analysis. *Optometry*.

[45] Heitger MH, Jones RD, Anderson TJ (2008). A new approach to predicting postconcussion syndrome after mild traumatic brain injury based upon eye movement function. *Conf. Proc. IEEE Eng. Med. Biol. Soc.*.

[46] Kraus MF, Little DM, Wojtowicz SM, Sweeney JA (2010). Procedural learning impairments identified via predictive saccades in chronic traumatic brain injury. *Cogn. Behav. Neurol.*.

[47] Galetta KM, Barrett J, Allen M (2011). The King-Devick test as a determinant of head trauma and concussion in boxers and MMA fighters. *Neurology*.

[48] Galetta KM, Brandes LE, Maki K (2011). The King-Devick test and sports-related concussion: study of a rapid visual screening tool in a collegiate cohort. *J. Neurol. Sci.*.

[49] King D, Brughelli M, Hume P, Gissane C (2013). Concussions in amateur rugby union identified with the use of a rapid visual screening tool. *J. Neurol. Sci.*.

[50] Galetta MS, Galetta KM, McCrossin J (2013). Saccades and memory: baseline associations of the King-Devick and SCAT2 SAC tests in professional ice hockey players. *J. Neurol. Sci.*.

[51] King D, Clark T, Gissane C (2012). Use of a rapid visual screening tool for the assessment of concussion in amateur rugby league: a pilot study. *J. Neurol. Sci.*.

[52] Leong DF, Balcer LJ, Galetta SL, Liu Z, Master CL (2014). The King-Devick test as a concussion screening tool administered by sports parents. *J. Sports Med. Phys. Fitness*.

[53] Leong DF, Balcer LJ, Galetta SL, Evans G, Gimre M, Watt D (2015). The King-Devick test for sideline concussion screening in collegiate football. *J. Optom.*.

[54] Dhawan P, Starling A, Tapsell L (2015). King-Devick test identifies symptomatic concussion in real-time and asymptomatic concussion over time. *J. Optom.*.

[55] Duenas M, Whyte G, Jandial R (2014). Sideline concussion testing in high school football on Guam. *Surg. Neurol. Int.*.

[56] King D, Gissane C, Hume PA, Flaws M (2015). The King-Devick test was useful in management of concussion in amateur rugby union and rugby league in New Zealand. *J. Neurol. Sci.*.

[57] Lin TP, Adler CH, Hentz JG, Balcer LJ, Galetta SL, Devick S (2014). Slowing of number naming speed by King-Devick test in Parkinson's disease. *Parkinsonism Relat. Disord.*.

[58] Moster S, Wilson JA, Galetta SL, Balcer LJ (2014). The King-Devick (K-D) test of rapid eye movements: a bedside correlate of disability and quality of life in MS. *J. Neurol. Sci.*.

[59] Ayaz H, Shewokis PA, Scull L (2014). Assessment of prefrontal cortex activity in amyotrophic lateral sclerosis patients with functional near infrared spectroscopy. *J. Neurosci. Neuroeng.*.

[60] Davies EC, Henderson S, Balcer LJ, Galetta SL (2012). Residency training: the King-Devick test and sleep deprivation: study in pre- and post-call neurology residents. *Neurology*.

[61] Stepanek J, Pradhan GN, Cocco D (2014). Acute hypoxic hypoxia and isocapnic hypoxia effects on oculometric features. *Aviat. Space Environ. Med.*.

[62] Stepanek J, Cocco D, Pradhan GN (2013). Early detection of hypoxia-induced cognitive impairment using the King-Devick test. *Aviat. Space Environ. Med.*.

[63] Liberati A, Altman DG, Tetzlaff J (2009). The PRISMA statement for reporting systematic reviews and meta-analyses of studies that evaluate healthcare interventions: explanation and elaboration. *BMJ*.

[64] Higgins JPT, Thompson SG, Deeks JJ, Altman DG (2003). Measuring inconsistency in meta-analyses. *BMJ*.

[65] Luna B, Velanova K, Geier CF (2008). Development of eye-movement control. *Brain Cogn.*.

[66] Bucci MP, Seassau M (2012). Saccadic eye movements in children: a developmental study. *Exp. Brain Res.*.

[67] Alahyane N, Brien DC, Coe BC, Stroman PW, Munoz DP (2014). Developmental improvements in voluntary control of behavior: effect of preparation in the fronto-parietal network?. *Neuroimage*.

[68] Radomski MV, Finkelstein M, Llanos I, Scheiman M, Wagener SG (2014). Composition of a vision screen for service members with traumatic brain injury: consensus using a modified nominal group technique. *Am. J. Occup. Ther.*.

[69] Singman EL (2013). Automating the assessment of visual dysfunction after traumatic brain injury. *Medical Instrumentation*.

[70] Beh SC, Frohman TC, Frohman EM (2014). Neuro-ophthalmic manifestations of cerebellar disease. *Neurol. Clin.*.

[71] Quinet J, Goffart L (2015). Cerebellar control of saccade dynamics: contribution of the fastigial oculomotor region. *J. Neurophysiol.*.

[72] Munce TA, Dorman JC, Odney TO, Thompson PA, Valentine VD, Bergeron MF (2014). Effects of youth football on selected clinical measures of neurologic function: a pilot study. *J. Child Neurol.*.

[73] Munce TA, Dorman JC, Thompson PA, Valentine VD, Bergeron MF (2014). Head impact exposure and neurologic function of youth football players. *Med. Sci. Sports Exerc.*.

[74] King D, Hume P, Gissane C, Clark T (2015). Use of the King-Devick test for sideline concussion screening in junior rugby league. *J. Neurol. Sci.*.

